# Interaction between *PNPLA3* I148M Variant and Age at Infection in Determining Fibrosis Progression in Chronic Hepatitis C

**DOI:** 10.1371/journal.pone.0106022

**Published:** 2014-08-29

**Authors:** Stella De Nicola, Paola Dongiovanni, Alessio Aghemo, Cristina Cheroni, Roberta D'Ambrosio, Michele Pedrazzini, Francesco Marabita, Lorena Donnici, Marco Maggioni, Silvia Fargion, Massimo Colombo, Raffaele De Francesco, Luca Valenti

**Affiliations:** 1 Department of Gastroenterology, Fondazione IRCCS Ca' Granda Ospedale Policlinico Milano, Milano, Italy; 2 Department of Internal Medicine, Fondazione IRCCS Ca' Granda Ospedale Policlinico Milano, Milano, Italy; 3 Virology Program, INGM - Istituto Nazionale di Genetica Molecolare “Romeo ed Enrica Invernizzi,” Milano, Italy; 4 Department of Pathophysiology and Transplantation, Università degli Studi di Milano, Milano, Italy; 5 Department of Pathology, Fondazione IRCCS Ca' Granda Ospedale Policlinico Milano, Milano, Italy; University of Navarra School of Medicine and Center for Applied Medical Research (CIMA), Spain

## Abstract

**Background and Aims:**

The *PNPLA3* I148M sequence variant favors hepatic lipid accumulation and confers susceptibility to hepatic fibrosis and hepatocellular carcinoma. The aim of this study was to estimate the effect size of homozygosity for the *PNPLA3* I148M variant (148M/M) on the fibrosis progression rate (FPR) and the interaction with age at infection in chronic hepatitis C (CHC).

**Methods:**

FPR was estimated in a prospective cohort of 247 CHC patients without alcohol intake and diabetes, with careful estimation of age at infection and determination of fibrosis stage by Ishak score.

**Results:**

Older age at infection was the strongest determinant of FPR (p<0.0001). *PNPLA3* 148M/M was associated with faster FPR in individuals infected at older age (above the median, 21 years; −0.64±0.2, n = 8 vs. −0.95±0.3, n = 166 log_10_ FPR respectively; p = 0.001; confirmed for lower age thresholds, p<0.05), but not in those infected at younger age (p = ns). The negative impact of *PNPLA3* 148M/M on fibrosis progression was more marked in subjects at risk of altered hepatic lipid metabolism (those with grade 2–3 steatosis, genotype 3, and overweight; p<0.05). At multivariate analysis, *PNPLA3* 148M/M was associated with FPR (incremental effect 0.08±0.03 log_10_ fibrosis unit per year; p = 0.022), independently of several confounders, and there was a significant interaction between 148M/M and older age at infection (p = 0.025). The association between 148M/M and FPR remained significant even after adjustment for steatosis severity (p = 0.032).

**Conclusions:**

We observed an interaction between homozygosity for the *PNPLA3* I148M variant and age at infection in determining fibrosis progression in CHC patients.

## Introduction

Hepatitis C virus (HCV) infection affects roughly 130–170 million people worldwide. Individuals with chronic HCV-related hepatitis (CHC) are at high risk of liver fibrosis, an in about 20% of cases CHC progresses to cirrhosis. Therefore, HCV is a leading cause of end-stage liver disease and hepatocellular carcinoma [1]. Older age at infection, genotype 3, coexistent liver diseases, male gender, alcohol abuse, obesity and diabetes, and steatosis are risk factors for disease evolution [2]–[5].

However, even after taking into account all known viral, host related, and environmental factors, the rate of progression to advanced fibrosis is highly variable among individuals, thereby suggesting a role for inherited variants. Indeed, single-nucleotide polymorphisms at different genetic loci have been associated with fibrosis progression in candidate gene studies [6], [7].

The rs738409 C>G polymorphism of *Patatin-like phospholipase domain-containing 3* (*PNPLA3*), encoding for the I148M protein sequence variant, is the major common genetic determinant of hepatic fat content. The I148M variant influences the metabolism of triglycerides, lipid droplets remodeling, and lipoprotein secretion in hepatocytes [8]–[13]. *PNPLA3* I148M is the major risk factor for both alcoholic and nonalcoholic steatohepatitis, showing an age-modulated effect on liver damage susceptibility and fibrogenesis [14]–[19]. Recently, it has been reported that homozygosity for the 148M risk allele (henceforth *PNPLA3* 148M/M) also influences the risk of advanced fibrosis and hepatocellular carcinoma in CHC [20]–[25]. However, scant data are derived from prospective studies [23], [25], and the interaction with age at infection has not been evaluated so far.

Therefore, the aim of this study was to estimate the effect size of *PNPLA3* 148M/M on fibrosis progression rate (FPR) and the interaction with age at infection in a unique validated cohort of 247 CHC subjects without significant alcohol intake and diabetes, characterized by a careful estimation of age at infection and with histological determination of liver damage [3].

## Materials and Methods

### Patients

We analyzed a previous published cohort of 247 CHC patients followed-up at the Migliavacca Liver Disease Center at the Fondazione IRCCS Ca' Granda Policlinico of Milan [3]. Briefly, inclusion criteria comprised a precise estimate of the date of infection based on the date of the first reported risk factor, a diagnostic liver biopsy before any antiviral therapy performed at least 4 years after the date of infection, and no history of past or current alcohol abuse. Patients with viral co-infections or other liver diseases were excluded, as well as patients with either type 1 or type 2 diabetes. Disease duration was calculated considering the time elapsed from the date of infection to the time of liver biopsy. The study protocol conformed to the Declaration of Helsinki and was approved by the Institutional Review Board of the Fondazione IRCCS Ca' Granda. Each patient signed a written informed consent.

The baseline features of the study cohort stratified by *PNPLA3* 148M/M status are presented in table 1. The study database has been uploaded as supplementary material (Data S1).

**Table 1:** Clinical features of 247 CHC patients subdivided according to the presence of homozygosity for *PNPLA3* I148M variant (148M/M).

	*PNPLA3* I148M genotype		p value
	148I/I or I/M	148M/M	
	n = 226 (91)	n = 21 (9)	
Age at infection years	21.5±13	21.7±16	0.96
Sex F	109 (48)	9 (43)	0.66
BMI Kg/m^2^	26.1±13	24.9±6	0.69
Age at biopsy years	46.8±12	48.4±13	0.70
HCV genotype			0.64
G1	116 (51)	13 (62)	
G2	68 (30)	6 (28)	
G3	32 (14)	2 (10)	
G4	10 (5)	0	
Route of infection			0.83
Transfusion	169 (75)	16 (76)	
IVDU	53 (23)	5 (24)	
Other	4 (2)	0	
Steatosis grade 2–3	42 (19)	5 (26)	0.48
Disease activity (grade)	6 {5–7}	6 {5–7}	0.78
Fibrosis stage	2 {1–3}	3 {2–5}	0.22

Continous variables are shown as mean ± SD (normally distributed) or median {interquartile range} (skewed variables); (): % values; F: female; BMI: body mass index; G: genotype; IVDU: intra-venous drug user; Other: needlestick, mother-to-child, sexual transmission.

### Histological evaluation

Liver histology was evaluated by a single expert pathologist. Mean biopsy length was 26.3 mm. The severity of hepatic inflammation was evaluated by the Ishak score [26], entailing a maximum of 18 points for grading (G) and 6 points for liver staging (S). All specimens were also evaluated for steatosis severity (grade 0: absent or <5%; grade 1: 5–33%; grade 2: 34–66%; grade 3: >66% of hepatocytes affected) [27]. Steatosis assessment is missing for 14 patients due to the inability to recollect the original histological slides re-evaluated for fibrosis stage. To analyze the effect of steatosis severity in the whole study cohort, additional analyses were conducted estimating the severity of steatosis in these 14 subjects by ultrasonography [28].

### Genetic analysis

Genomic DNA was obtained from peripheral whole blood as previously described [3]. The *PNPLA3* rs738409 C>G single nucleotide polymorphism, encoding for the I148M protein sequence variant, was genotyped by a 5^′-^nuclease Taqman assay (LifeTechnologies, Carlsbad, CA, USA) at the Metabolic Liver Diseases lab at the Fondazione Ca' Granda IRCCS. Success rate was 100% and perfect concordance between duplicates and internal controls was observed.

### Statistical analysis

For descriptive statistics, variables were shown as mean ± SD and frequencies, and compared by Student's t-test or chi-square test according to data distribution. P values were considered significant when <0.05 (two-tailed). FPR was calculated by taking the ratio between the staging value of Ishak score and the disease duration (years), and it was treated as a continuous variable. Mean disease duration was 25±10 years. Log_10_ transformation of FPR was employed to obtain linearity. FPR estimation assumes that no significant liver fibrosis was present at the time of infection, and that liver fibrosis progression is constant during time [2]. A first generalized linear model was formulated and fitted to the data, specifying the presence of *PNPLA3* 148M/M at risk genotype [21]–[23], and the covariates as explanatory variables. We considered as covariated the independent predictors of FPR in a previously reported analysis of the same cohort [3]: age at infection (encoded as > or ≤ the median value, 21 years), gender, and HCV genotype. The product term age at infection x *PNPLA3* 148M/M was fitted into the model to test the interaction between *PNPLA3* genotype and age at infection on FPR. To account for other potential confounders, a second model was tested, with further adjustement for the route of HCV transmission (intra-venous drug use: IVDU or other), body-mass (BMI < or ≥25 Kg/m^2^), and age at liver biopsy. Finally, to check whether the relationship between *PNPLA3* genotype and FPR was independent of steatosis, a final model further adjusted for the histological severity of steatosis (grade 2–3 vs. 0–1) was evaluated. Models were checked through the regression diagnostic plots to verify normality, linearity of the data, and constant variance. The effect of the explanatory variables was considered significant if p<0.05 (two-tailed). Analyses were performed by the SPSS 21.0 (IBM, Burbank, NY, USA) statistical analysis software.

## Results

### Study cohort

Detailed characterization of the study cohort has previously been reported [3]. Briefly, 52% of patients were males, the majority infected by genotype 1 (52%), during blood transfusion (75%). Median age at infection was 21 years and disease duration 25 years. At the time of histological evaluation, 16% of patients had cirrhosis, whereas severe steatosis (grade 2–3) was observed in 20% of cases. The frequency distribution of the *PNPLA3* I148M variant did not violate Hardy-Weinberg equilibrium (p>0.8), and was in line with that expected unselected subjects: 120 patients were *PNPLA3* 148I/I (48%), 106 148I/M (43%), and 21 had the unfavorable 148M/M genotype (9%).

Clinical features of patients stratified by *PNPLA3* 148M/M status are presented in table 1. Demographic and anthropometric features, viral genotype distribution, age and route of infection, and duration of follow-up did not differ according to *PNPLA3* 148M/M status (p = ns). There was no statistically significant difference in the prevalence of grade 2–3 steatosis according to *PNPLA3* 148M/M status (p = ns), although a nonsignificant trend was observed in the whole cohort including subjects with ultrasonographic estimation of steatosis severity (7/21, 33% vs. 44/226, 19% in 148M/M vs. individuals carrying other *PNPLA3* genotypes, p = 0.15).

### Effect of age at infection and *PNPLA3* 148M/M on fibrosis progression

**Figure 1:**
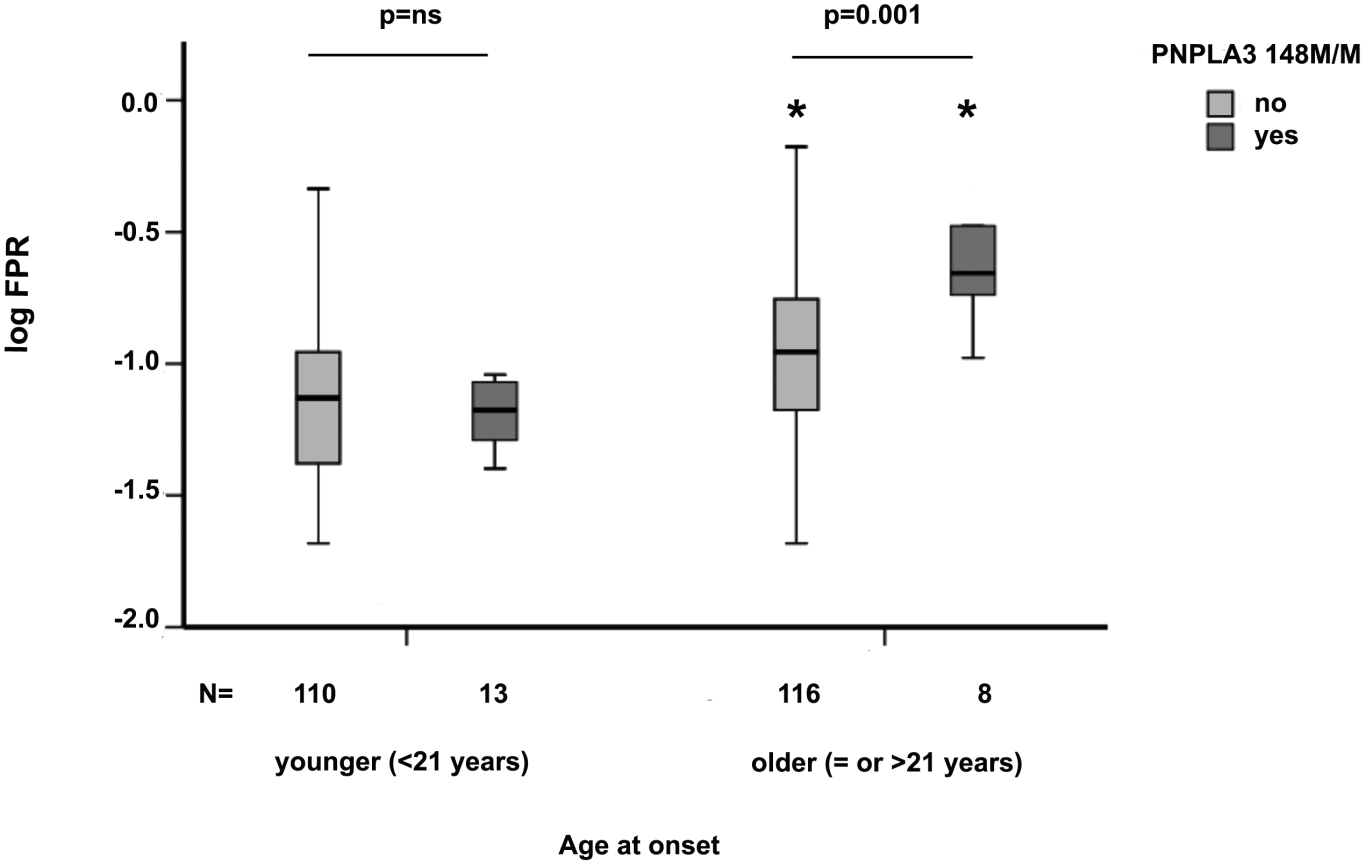
Fibrosis progression rate (FPR) according to age at infection (> or ≤21 years, median value) and *PNPLA3* 148M/M status in 247 patients with chronic hepatitis C. Box plots indicate median value and interquatile ranges, whiskers the 5^th^ and 95^th^ centiles. p = 0.001 for faster FPR in *PNPLA3* 148M/M positive vs. negative patients with older age at infection (>21 years). *p<0.0001 vs. patients with same *PNPLA3* 148M/M status and younger age at infection (≤21 years).

Older age at infection was strongly associated with FPR (log_10_ FPR −1.1±0.3 vs. −0.9±0.3 in subjects younger or older than 21 years, median value, respectively; p<0.0001). The FPR stratified by age at infection (> or ≤ median value, 21 years) and *PNPLA3* 148M/M and is presented in figure 1 and in table 2. *PNPLA3* 148M/M was associated with faster FPR in older (p = 0.001), but not in younger patients (p = ns). The positive association between *PNPLA3* 148M/M and FPR in older patients was confirmed in sensitivity analyses using different age cut-offs (table 2): 15 years, the 25^th^ centile (p = 0.038), 18 years (p = 0.045), and 27 years, the 75^th^ centile (p = 0.01).

**Table 2:** Effect of PNPLA3 148M/M on (log_10_) FPR in 247 CHC patients according to different thresholds of age at infection.

	PNPLA3		p value
	148I/I or I/M	148M/M	
15 years; 25^th^ centile (±puberty)			
Younger	−1.25±0.3 (51)	−1.19±0.3 (9)	ns
Older	−0.98±0.3 (175)	−0.78±0.3 (12)	0.038
18 years; “adulthood”			
Younger	−1.25±0.3 (70)	−1.19±0.3 (9)	ns
Older	−0.99±0.3 (156)	−0.78±0.3 (12)	0.045
21 years; median			
Younger	−1.14±0.3 (110)	−1.16±0.3 (13)	ns
Older	−0.95±0.3 (116)	−0.64±0.2 (8)	0.001
27 years; 75^th^ centile			
Younger	−1.10±0.3 (157)	−1.11±0.3 (14)	ns
Older	−0.90±0.3 (69)	−0.67±0.2 (7)	0.01

(): number of subjects; ns: not significant.

### Steatogenic factors, *PNPLA3* 148M/M, and fibrosis progression

Moderate-severe steatosis was associated with faster FPR (log_10_ FPR −1.1±0.3 vs. −0.9±0.3 in subjects with steatosis grade 0–1, n = 186 vs. grade 2–3, n = 47, respectively; p = 0.016). The association between steatosis grade 2–3 and FPR was confirmed in the whole cohort including subjects with ultrasonographic estimation of steatosis severity (log_10_ FPR −1.1±0.3 vs. −0.9±0.3 in subjects with steatosis grade 0–1, n = 196 vs. grade 2–3, n = 51, respectively; p = 0.012). Since PNPLA3 I148M interferes with hepatocellular lipid metabolism, we next evaluated whether the effect of *PNPLA3* 148M/M on FPR was influenced by steatosis severity and risk factors for derangement of hepatic lipid metabolism. Results are shown in table 3. The association of *PNPLA3* 148M/M with FPR was more consistent in the presence of grade 2–3 steatosis (p = 0.024), or in the presence of other steatogenic factors: namely infection with genotype 3 (p = 0.005), or overweight (p = 0.05). The association between *PNPLA3* 148M/M and FPR was nearly significant in patients with steatosis grade 2–3 including subjects with ultrasonographic estimation of steatosis severity (p = 0.056).

**Table 3:** Effect of PNPLA3 148M/M on (log_10_) FPR in 247 CHC patients according to steatosis severity and the presence of risk factors for steatosis/altered hepatic lipid metabolism (HCV-G3 and overweight).

	PNPLA3		p value
	148I/I or I/M	148M/M	
Steatosis grade			
0–1	−1.06±0.3 (172)	−1.07±0.3 (14)	ns
2–3	−0.97±0.3 (42)	−0.67±0.2 (5)	0.024
HCV genotype			
G1, G2, G4	−1.06±0.3 (194)	−1.00±0.3 (19)	ns
G3	−0.92±0.3 (32)	−0.70±0.0 (2)	0.005
Overweight			
BMI≤25 Kg/m^2^	−1.01±0.3 (116)	−1.13±0.3 (8)	ns
BMI>25 Kg/m^2^	−1.07±0.3 (110)	−0.87±0.2 (13)	0.05

(): number of subjects; BMI: body mass index; ns: not significant.

### Independent predictors of fibrosis progression

At the multivariate generalized linear model considering known determinants of FPR (age at infection, gender, and viral genotype: shown in model 1, table 4) *PNPLA3* 148M/M was an independent predictor of FPR (p = 0.022). The model estimated an incremental progression of 0.08±0.03 log_10_ unit of fibrosis per year conferred by 148M/M status. Furthermore, there was a significant interaction between *PNPLA3* 148M/M and older age at infection in determining FPR (p = 0.025; estimate +0.07±0.03). In exploratory analyses, there was no significant interaction between *PNPLA3* 148M/M and other determinants of FPR.

**Table 4:** Independent determinants of (log_10_) FPR at multivariate generalized linear models in 247 CHC patients.

	Model 1			Model 2			Model 3
	estimate	SE	p value	estimate	SE	p value	estimate	SE	p value
Sex F	−0.05	0.02	0.005	−0.05	0.02	0.006	−0.05	0.02	0.01
Older age at infection	+0.21	0.03	<0.0001	+0.21	0.03	<0.0001	+0.21	0.04	<0.0001
HCV genotype									
G1	ref			ref			ref		
G2	−0.10	0.04	0.004	−0.11	0.03	0.003	−0.10	0.04	0.008
G3	+0.13	0.04	0.002	+0.13	0.04	0.004	+0.11	0.05	0.02
G4	0.00	0.06	>0.1	0.02	0.06	>0.1	0.02	0.07	>0.1
*PNPLA3* 148M/M	+0.08	0.03	0.022	+0.08	0.03	0.018	+0.07	0.03	0.032
*PNPLA3* 148M/M * older age at infection	+0.07	0.03	0.025	+0.07	0.03	0.021	+0.07	0.03	0.045
Age at biopsy	NA			−0.003	0.002	0.06	−0.003	0.002	0.06
BMI>25 Kg/m^2^	NA			−0.02	0.02	>0.1	−0.02	0.02	>0.1
IVDU	NA			−0.02	0.05	>0.1	−0.01	0.05	>0.1
Steatosis grade 2–3 vs. 0–1	NA			NA			+0.04	0.02	0.03

NA: not addressed; F: female; BMI: body mass index; IVDU: intravenous drug use.

Model 1: minimal model considering previously identified risk factors for FPR in the present cohort [3], plus 148M/M and the interaction between 148M/M and age infection. Model 2: extended model further corrected for potential confounding factors. Model 3: extended model with additional correction for histological severity of steatosis. P values are shown for ≤0.1.

To test the robustness of the association between *PNPLA3* genotype and FPR, we extended the model with further adjustment for additional potential confounders: age at biopsy, presence of overweight, and route of infection (model 2; table 4). In this extended model, the association of *PNPLA3* 148M/M with FPR (estimate +0.08±0.03, p = 0.018), as well as the interaction with older age at infection (estimate +0.07±0.03, p = 0.021) remained unaffected.

Finally, to test whether the effect of 148M/M status on fibrosis progression was independent of the histological severity of steatosis, we further adjusted the extended model for the presence of grade 2–3 steatosis (model 3; table 4). Again, *PNPLA3* 148M/M (estimate +0.07±0.03, p = 0.032) and the interaction with older age at infection (estimate +0.07±0.03, p = 0.045) remained significantly associated with FPR independently of the steatosis severity (grade 2–3 vs. 0–1; estimate +0.04±0.02, p = 0.03). In the overall series of patients including those with ultrasonographic estimation of steatosis, *PNPLA3* 148M/M (estimate +0.07±0.03, p = 0.033) and the interaction with older age at infection (estimate +0.07±0.03, p = 0.036) remained significantly associated with FPR.

## Discussion

In this paper, we showed that in a prospective cohort with careful estimation of the date of infection and absence of major confounders the *PNPLA3* I148M sequence variant influences fibrosis progression in CHC.

Homozygosity for the I148M variant, which in line with the prevalence in the Italian population [29] was detected in about one in ten subjects, was associated an average incremental progression of fibrosis stage of as much as circa 0.8 units of Ishak score per year. The link between 148M/M status and FPR was independent of known moderators of fibrogenesis in CHC [2], [3], [30], and remained robust even after extensive adjustment for other potential confounders.

Our study is the first to show that the effect of *PNPLA3* I148M on fibrosis progression in CHC is modulated by the age at infection. The negative impact of 148M/M on FPR in individuals infected at older age was confirmed in sensitivity analyses with lower age cut-offs, and was prominent in individuals infected in adulthood. Previous studies suggested that *PNPLA3* I148M modulates liver damage progression in an age-dependent fashion. However, despite older age at onset was associated with faster progression of liver damage in alcoholic liver disease, the effect size of 148M/M was larger in younger patients [19]. Similarly, the reported effect size of the *PNPLA3* I148M variant on liver damage in nonalcoholic fatty liver disease was larger in pediatric case series [14]–[16].

The reason underlying the opposite direction of modulation by age of the effect of *PNPLA3* I148M on fibrosis progression in CHC is presently unknown. It could be speculated that the pattern of activation of the immune system towards HCV is different depending on the age at infection. According to this hypothesis, HCV infection after the developmental age would lead to immune-mediated hepatic damage, exerting a permissive role on *PNPLA3* I148M-related fibrogenesis [31]. Alternatively, older age may synergize with *PNPLA3* I148M in determining hepatic steatosis, which would favor viral replication during the acute infection [32], thus setting the stage for faster disease progression.

Indeed, evidence is accumulating that *PNPLA3* I148M synergizes with host features and environmental triggers in determining liver damage associated with altered hepatic lipid metabolism [33]. In line with these data, we found that the effect of *PNPLA3* 148M/M on fibrogenesis was more evident in subjects with grade 2–3 steatosis, and in those with other conditions favoring hepatocellular lipid accumulation, such as infection with genotype 3 and overweight.

Of note, we reported for the first time an effect of 148M/M on fibrosis progression in genotype 3 patients, even if previous studies did not detect an association between *PNPLA3* and steatosis in this subgroup [34], [35]. However, since this finding was based on FPR assessment in only two 148M/M patients infected with HCV G3, it will require confirmation in future studies. Most importantly, the effect of 148M/M on fibrogenesis was independent of the histological severity of steatosis. These data are in line with results of cross-sectional studies in nonalcoholic fatty liver disease [33], and suggest that *PNPLA3* I148M may promote fibrogenesis by directly altering hepatic stellate cells lipid metabolism and trans-activation [36].

Therefore, given the independent association of the *PNPLA3* I148M variant with fibrosis progression and hepatocellular carcinoma risk [24], *PNPLA3* I148M genotyping may help refining treatment prioritization to novel therapeutic regimens based on direct antiviral agents [37], and patients follow-up.

Limitations of this study include the assumption of the absence of liver fibrosis at the time of infection, which is likely given the absence of cofactors of liver damage, the young age at infection and low risk profile. Furthermore, the model tested assumes the consistency and linearity of FPR during time, and of fibrosis progression across Ishak score stages, which are approximations of a more complex reality [38]. Notwithstanding, the present results are corroborated by the fact that our approach was able to validate the association of FPR with the major risk factors for fibrosis progression in CHC [3], and by independent findings linking *PNPLA3* 148M/M with fibrogenesis in CHC [25], [33].

## Conclusions

In conclusion, homozygosity for the *PNPLA3* I148M variant is associated with FPR in CHC. There is a significant interaction between age at infection and *PNPLA3* genotype in determining fibrosis progression, but the association between *PNPLA3* and FPR is independent of the histological severity of steatosis. These findings further establish the *PNPLA3* I148M variant as a moderator of CHC natural history, and have possible relevance for treatment prioritization and follow-up of CHC patients.

## Supporting Information

Data S1 **Study database.**
(XLSX)
